# Real-world evidence in localized and locally advanced prostate cancer: applying artificial intelligence to electronic health records

**DOI:** 10.1186/s12885-025-14828-z

**Published:** 2025-10-21

**Authors:** José Pablo Maroto, Javier Puente, Antonio Conde Moreno, Álvaro Juárez, Beatriz Garcillán, José Miguel Calderón, Jacobo Muñoz del Toro, Juan Luis Valdivieso, María López-Menduiña, Eduard Sarró, Antonio Alcaraz

**Affiliations:** 1 Department of Medical Oncology, Hospital de La Santa Creu I Sant Pau, Barcelona, Spain; 2 Department of Medical Oncology, Hospital Clínico San Carlos, Madrid, Spain; 3 Department Of Radiation Oncology, Hospital Universitario La Fe, Valencia, Spain; 4 Department of Urology, Hospital Universitario de Jerez, Jerez, Spain; 5 Medical Affairs Department, Janssen-Cilag S.A. a Johnson & Johnson Company, Madrid, Spain; 6 Savana Research S.L., Madrid, Spain; 7 Department of Urology, Hospital Clinic, Barcelona, Spain

**Keywords:** Prostate cancer, Localized and locally advanced prostate cancer, Electronic health records, Natural language processing, Machine learning

## Abstract

**Purpose:**

To provide real-world evidence of the clinical characteristics and outcomes of localized and locally advanced prostate cancer patients (LPC/LAPC).

**Materials & methods:**

Observational and retrospective analysis using secondary data from electronic health records (EHR) of prostate cancer (PC) patients in eight Spanish hospitals (2014–2018). Data was extracted and analyzed using EHRead® technology, based on natural language processing and machine learning. LPC/LAPC patients were included and stratified by risk and by first treatment received.

**Results:**

Twenty-two thousand one hundred sixty-six PC patients were identified,14,434 (65.1%) were classified as LPC/LAPC. Among them, 5,331 incident patients with sufficient data were selected for outcome analysis (real world overall survival [rwOS], metastasis and event free survival [MFS, EFS]) and were followed for a median time of 2.3 years. 36.5% were classified as LPC intermediate risk (IR), 26.0% LPC high risk (HR), 7.3% LPC low risk (LR), 5.9% LAPC, and 24.2% unknown risk. First treatment received was radiotherapy (RT) in 40.7%, radical prostatectomy (RP) in 37.1%, active surveillance (AS)/watchful waiting (WW) in 6.4%, brachytherapy (BT) in 4.2%, and androgen deprivation therapy monotherapy (ADT only) in 3.3%. rwOS and MFS worsened as risk increased. Patients treated with ADT only presented the worst baseline characteristics, showing limited clinical outcomes. The 36-month rwOS was 91% for LAPC patients, 93% for HR-LPC, 97% for IR-LPC, and 98% for LR-LPC.

**Conclusions:**

Despite using treatment with curative intent, patients experienced oncological events within a median of less than three years post-diagnosis. Our findings emphasize the need for risk stratification, and proactive strategies to improve clinical outcomes.

**Supplementary Information:**

The online version contains supplementary material available at 10.1186/s12885-025-14828-z.

## Introduction

Prostate cancer (PC) is the most commonly diagnosed cancer among European males and the third leading cause of cancer death in men in Europe [1]. At the time of diagnosis, a large percentage of patients (> 80%) present with localized organ-confined disease (localized PC or LPC). Locally advanced PC (LAPC) with invasion of some periprostatic tissue and local lymph nodes is reported in 10–15% of patients, according to surgical series involving patients undergoing radical prostatectomy (RP) [2, 3].

Current guidelines from the European Association of Urology (EAU) for managing LPC/LAPC are based on risk stratification [4]. Watchful waiting (WW) is recommended for patients with a life expectancy of less than 10 yr. Active surveillance (AS) is advised for men with low metastatic potential to mitigate overtreatment, meanwhile local therapy is reserved for patients with a life expectancy over 10 years and with risk of recurrence. Importantly, ADT monotherapy should only be offered to high-risk (HR)-LPC patients who are unable to receive any form of local therapy [4]. A recent survey of Spanish urologists and oncologists found that radical treatment was used in 58% of low-risk, 91% of intermediate-risk, and 86% of high-risk PC cases, while active surveillance (AS) was applied in 38%, 2%, and 1%, respectively and ADT in monotherapy in 1%, 6%, and 13%, respectively [5]. Although overall PC-specific mortality is low regardless of the treatment assigned, management of PC in daily practice is still a challenging endeavor for clinicians [3].

The potential of artificial intelligence (AI) has been explored in understanding and treating various diseases, including PC [6–10]. AI aids in diagnosis, risk prediction, and treatment decisions with accuracy comparable to that of field experts [11, 12]. Among AI applications, natural language processing (NLP) and machine learning (ML) are especially promising for extracting critical information from electronic health records (EHRs) [13, 14]. While structured fields (e.g., diagnostic codes) are commonly used, they often lack the clinical depth required for comprehensive patient characterization, and in settings like Spain, coding is not systematically completed across institutions [15, 16]. Unstructured free-text notes such as clinical visit summaries, imaging or pathology reports, contain richer and more nuanced data that reflect actual clinical reasoning and patient trajectories [17]. Although NLP has been used in some studies to complement structured data, relying primarily on clinical NLP trained on large corpora of medical text enables more representative and scalable real-world data extraction. This approach supports deeper understanding of real-life clinical pathways and facilitates the development of tools that better inform personalized therapeutic strategies [18].

This sub-study is part of the wider *OVERVIEW Study* (“An observational study to retrospectively describe the evolution of patients with prostate cancer throughout the disease period by using electronic health records with artificial intelligence”), which investigates the epidemiology, diagnosis, management, and outcomes of PC patients in Spain’s real-world clinical setting using clinical NLP and ML techniques. Beyond its descriptive purpose, the OVERVIEW study highlights the potential of large-scale, AI-driven extraction of unstructured EHR data as a foundational step toward developing next-generation registries and enabling future real-time monitoring and planning within healthcare systems. Here, we aimed to identify and describe LPC/LAPC patients, characterizing them in terms of demographic and clinical features, treatment pathways, health care resource utilization as well as to evaluate clinical outcomes stratifying by risk and by first treatment received.

## Materials and methods

### Study design and data source

**Fig. 1:**
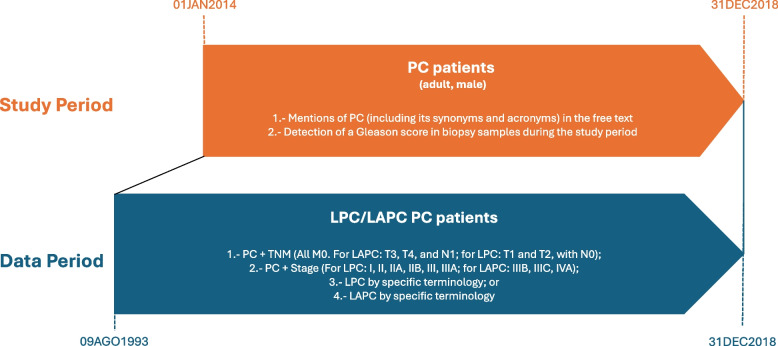
Study design. This figure illustrates the study period (01JAN2014–31DEC2018) during which PC patients were identified based on (1) free-text mentions of PC (including synonyms and acronyms) and (2) detection of a Gleason score in biopsy samples. A broader data period (09AUG1993–31DEC2018) was used to characterize patients classified as having LPC/LAPC, based on available information on TNM staging, clinical stage, or specific terminology extracted from EHRs. The methodological framework allowed for accurate retrospective classification and analysis of patients using both structured and unstructured data. PC: prostate cancer; LPC: localized prostate cancer; LAPC: locally advanced prostate cancer

The OVERVIEW study was a multicenter, observational study launched in 2019, based on the secondary use of data retrospectively extracted from unstructured and, when available, structured text within the EHRs of adult patients with a PC diagnosis between January 1, 2014, and December 31, 2018 (study period). In addition, longitudinal information prior to 2014 was used when available, with the earliest records dating back to August 9, 1993, a range defined as the data period (Fig. 1).

Clinical information was processed and extracted using NLP and ML techniques, applied to EHRs collected across eight Spanish hospitals participating in the study (Supplementary Table S1). PC was ascertained based on either the presence of PC-related terms in the free-text data or the detection of a Gleason score (GS) in any reports during the study period.

### Study population

This sub-study presents data from patients diagnosed with LPC/LAPC according to the EAU classification [19], with categorization criteria outlined in Supplementary Methods and Fig. 1. Outcomes were stratified by first treatment received and D'Amico risk groups [20]. For these analyses, additional filters were applied to ensure a well-defined cohort: 1) first mention of LPC/LAPC within the study period (to ensure the inclusion of incident patients); 2) available GS and prostate specific antigen (PSA) at inclusion (to allow for accurate risk stratification); and 3) follow-up time different to zero (to ensure sufficient outcome data).

Patients were included in a cross-sectional analysis at index date, defined as the first time point during the data period at which the patient was identified as LPC/LAPC. Additionally, longitudinal analyses of the follow-up period were also performed. The follow-up period was defined as the time between the index date and the last available EHR (Supplemental methods).

### Stratifications

Patients were stratified based on the first treatment received after the index date and classified into the following groups: radiotherapy (RT), RP, brachytherapy (BT), AS, WW, ADT only, other treatment, and no treatment identified. Both AS and WW were identified through explicit mentions in free text or inferred from specific terms (e.g., “active surveillance,” “observation,” or “expectant management”) and the absence of active treatment within six months of diagnosis. If any treatment was initiated within that period, AS was disregarded, and the latter considered the actual first intervention. Although AS and WW represent different clinical approaches, they were grouped together (AS/WW) for the purposes of analysis, as both reflect strategies that do not involve immediate active procedures or specific treatments. The “ADT only” group included patients receiving ADT and who had no recorded local curative treatments (e.g., RP, RT, BT) either at treatment initiation or during the entire period under the LPC diagnosis. The other category included patients who: i) received less common local therapies; or ii) had multiple treatments detected as the initial intervention but without a clear sequence to determine which was first. Patients with no treatment identified were excluded from further analyses.

Risk stratifications were defined as low risk LPC (LR-LPC), intermediate risk LPC (IR-LPC), high risk LPC (HR-LPC), LAPC or as unknown risk (non-categorizable risk). Patients were stratified into D’Amico risk groups based on PSA levels, clinical staging, and Gleason scores, or based on terminology used in the EHR when structured data were unavailable. Further details are provided in the Supplementary Methods.

### Data source and data extraction

The primary data source was unstructured free text within the EHRs, complemented by structured data when available. Data were collected from all available departments in each participating site (including inpatient hospital, outpatient hospital, and emergency service). Clinical data was extracted using EHRead® (Medsavana S.L., Madrid, Spain) technology following previously described methods [21]. This data-driven methodology relies on NLP and ML using Systematized Nomenclature of Medicine–Clinical Terms (SNOMED CT) terminology to generate a synthetic database. Specifically, the extraction was performed using a clinical NLP system fully trained on real-world clinical texts, optimized to interpret narrative content across multiple departments and institutions. The study population was filtered based on predefined terminology and rules, capturing the detection of specific medical concepts and associated metadata for their characterization. EHRead® performance was externally validated as previously described [22]. Further details on the EHRead® technology, including its reading performance, are provided in the Supplemental methods and Supplementary Table S2.

### Study variables and outcomes

In this study, we analyzed a comprehensive set of real-world variables, the majority of which were extracted from unstructured free-text clinical notes using NLP techniques and curated by medical experts, to identify and characterize patients with LPC and LAPC (see Supplemental methods for further details). These included demographic information, clinical features, treatment patterns, healthcare resource utilization, and clinical outcomes. Healthcare use domain included variables related to all-cause resource use, irrespective of whether the encounters were directly linked to PC, based on the rationale that the overall burden of illness (not only disease-specific care) is critical when evaluating real-world patient trajectories. For consistency across patients with varying follow-up durations, healthcare resource use was expressed as rates per 100 patient-years. Real world overall survival using oncology EHRs (rwOS) was identified by recorded deaths in the EHRs or assumed in the circumstances listed in Supplemental methods as previously described [23, 24]. Metastasis free survival (MFS) was defined as time from diagnosis until occurrence of distant metastasis. Event free survival (EFS) only included patients with event presentation after inclusion date. The definitions of the events are provided in Supplemental methods.

### Statistical analysis

The prevalence and incidence of LPC/LAPC were standardized by age using the European standard population (Eurostat 2013) [25]. The analysis included expressing categorical variables as frequencies and percentages, while continuous variables were described using the mean and standard deviation (SD) or median and interquartile range (Q1, Q3). Baseline characteristics across treatment groups were compared using ANOVA for continuous variables and chi-squared tests for categorical variables. Clinical outcomes were analyzed using Kaplan–Meier and Cox proportional hazards models. Reverse Kaplan–Meier estimation was applied to determine the distribution of follow-up times. The statistical significance threshold was set at *p* < 0.05, with adjustments for multiple hypothesis testing made using the Benjamini & Hochberg method. Comparisons were included to support descriptive interpretation and should not be considered formal inferential analyses, given the observational nature of the study and the absence of multivariable adjustment. All analyses were performed using R software (v4.0.2).

## Results

### Patients’ demographics, disease characteristics and management

**Fig. 2:**
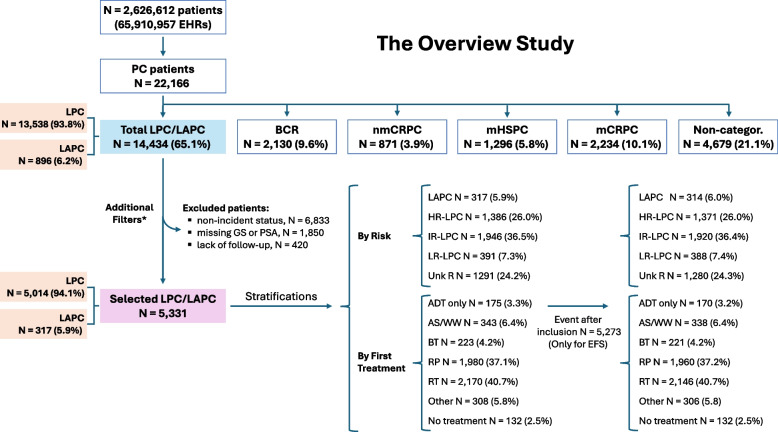
Flowchart of patient selection process and applied filters. For PC classification, please note that patients may fall into more than one subgroup. A total of 9,103 patients were excluded for outcome analyses. Three filters were sequentially applied to generate the final cohort, as shown in the Figure. * Patients could meet more than one exclusion criterion and from the total excluded population, 6,833 patients (47.3%) lacked incident LPC/LAPC classification, 3,793 (26.3%) lacked either Gleason score (2,669 patients), PSA (169 patients) or both (955 patients) at index date, and 543 (3.8%) had no follow-up data after diagnosis. ADT only group (*n* = 175) included 147 patients with only ADT mentions and 28 with ADT and AS mentions. PC: prostate cancer; LPC/LAPC: localized PC/locally advanced PC; BC: biochemical recurrence; nmCRPC: non-metastatic castration-resistant PC; mHSPC: metastatic hormone-sensitive PC; mCRPC: metastatic castration-resistant PC; Non-categor.: non-categorizable patients; LR: low risk; IR: intermediate risk; HR: high risk; Unk risk: unknown risk; RP: radical prostatectomy; RT: radiotherapy; BT: brachytherapy; AS/WW: Active surveillance/Watchful waiting; ADT: androgen deprivation therapy

From a population of 2,626,612 patients attended at the participating hospitals (totaling 65,910,957 EHRs) during the study period, 22,166 PC patients were identified (Fig. 2). Within the OVERVIEW study, these were classified into the following clinical stages: 65.1% LPC/LAPC (*n* = 14,434), 9.6% biochemical recurrence (*n* = 2,130), 3.9% non-metastatic castration-resistant PC (*n* = 871), 5.8% metastatic hormone-sensitive PC (*n* = 1,296), 10.1% metastatic castration-resistant PC (*n* = 2,234), and 21.1% non-categorizable patients (*n* = 4,679). Note that percentages do not add up to 100% as patients could be assigned to more than one clinical stage over time. For the present analysis, we focused on the LPC/LAPC subgroup. Demographic and clinical characteristics of these patients are shown in Supplementary Table S3 and S4. In this broader LPC/LAPC population, the age standardized prevalence was 729.1 per 100,000 persons in 2014 and increased to 895 per 100,000 persons in 2018. Conversely, incidence decreased from 123.3 to 91.9 per 100,000 persons per year between 2014 and 2018, respectively.

Clinical outcomes were analyzed in 5,331 LPC/LAPC selected patients meeting the following criteria: incident LPC/LAPC within the study period, available GS and PSA, and available follow-up. These filters resulted in the exclusion of 9,103 patients in total: 6,833 (75.1%) due to diagnosis outside the study period (i.e., non-incident cases), 1,850 (20.3%) due to missing GS or PSA, and 420 (4.6%) due to lack of follow-up, noting that patients could meet more than one exclusion criterion (Fig. 2 and Supplementary Figure S1). Characteristics of non-selected patients are shown in Supplementary Tables S3, S4 and S7. Median (Q1, Q3) age was 68 yr (63, 73), and 14.5% had family history of PC. Cardiovascular comorbidity was the most frequently reported, particularly hypertension in 41.6% and hypercholesterolemia in 28.2%. 67.8% of patients were asymptomatic based on the absence of symptom detection in EHRs (Table 1). Patients were stratified by first treatment received, 40.7% RT, 37.1% RP, 6.4% AS/WW, 4.2% BT, 3.3% ADT only (Table 1). A total of 308 (5.8%) patients fell into the “Other” category, comprising less common treatment modalities (Supplementary Table S5) whilst 132 (2.5%) had no treatment recorded. Median (Q1, Q3) PSA at diagnosis was 6.64 (4.50–10.83) ng/mL. Digital rectal examination was available for 45.2% of patients and 56.8% underwent imaging, with magnetic resonance imaging (MRI) performed in 61.1% patients. Genomic testing was performed in only 1.9% of patients, of which 3% were BRCA1 mutated and 7.1% BRCA2. Biopsy was ascertained in 98.0% of patients. Risk group distribution was as follows, 7.3% low-risk (LR)-LPC, 36.5% intermediate risk (IR)-LPC, 26.0% HR-LPC, 5.9% LAPC, and unknown risk was established in 24.2% of cases (Table 2).

**Table 1:** Demographic and clinical traits of LPC/LAPC patients at inclusion, overall and by initial treatment

	**Selected LPC/LAPC** ***n* = 5331**	**RP** ***n* = 1980**	**RT** ***n* = 2170**	**BT** ***n* = 223**	**AS/WW** ***n* = 343**	**ADT only** ***n* = 175**	**Other** ***n* = 308**	***p*-value**
Age, years, median (Q1, Q3)	68 (63, 73)	66 (61, 70)	71 (66, 75)	66 (60, 71.5)	69 (62, 74)	77 (69, 81)	67 (61, 71)	< 0.001*^&^
Age groups n (%)								
< 65 years	1668 (31.3)	844 (42.6)	426 (19.6)	97 (43.5)	121 (35.3)	21 (12)	121 (39.3)	< 0.001*
65–75 years	2783 (52.2)	990 (50)	1252 (57.7)	110 (49.3)	161 (46.9)	62 (35.4)	151 (49)	< 0.001*
> 75 years	880 (16.5)	146 (7.4)	492 (22.7)	16 (7.2)	61 (17.8)	92 (52.6)	36 (11.7)	< 0.001*
Family history of PC, n (%)	775 (14.5)	362 (18.3)	248 (11.4)	13 (5.8)	46 (13.4)	25 (14.3)	63 (20.5)	< 0.001*
Current smokers, n (%)	968 (18.2)	379 (19.1)	437 (20.1)	23 (10.3)	50 (14.6)	28 (16)	38 (12.3)	0.002*
Alcohol use, n (%)	399 (7.5)	135 (6.8)	190 (8.8)	9 (4)	28 (8.2)	12 (6.9)	22 (7.1)	0.046*
Main comorbidities, n (%)								
Hypertension	2219 (41.6)	732 (37)	988 (45.5)	77 (34.5)	165 (48.1)	98 (56)	108 (35.1)	< 0.001*
Hypercholesterolemia	1505 (28.2)	535 (27)	665 (30.6)	49 (22)	108 (31.5)	53 (30.3)	69 (22.4)	0.014*
Diabetes mellitus	917 (17.2)	324 (16.4)	395 (18.2)	27 (12.1)	60 (17.5)	44 (25.1)	40 (13)	0.010*
Chronic liver dysfunction	501 (9.4)	196 (9.9)	196 (9)	10 (4.5)	35 (10.2)	22 (12.6)	28 (9.1)	< 0.001*
Chronic obstructive pulmonary disease	411 (7.7)	113 (5.7)	209 (9.6)	9 (4)	23 (6.7)	26 (14.9)	21 (6.8)	< 0.001*
Obstructive sleep apnea	372 (7.0)	144 (7.3)	162 (7.5)	7 (3.1)	24 (7)	13 (7.4)	15 (4.9)	0.230
Cerebrovascular event	313 (5.9)	89 (4.5)	154 (7.1)	12 (5.4)	22 (6.4)	18 (10.3)	12 (3.9)	0.056
Chronic kidney disease	295 (5.5)	93 (4.7)	123 (5.7)	9 (4)	28 (8.2)	24 (13.7)	10 (3.2)	0.002*
Myocardial infarction	261 (4.9)	68 (3.4)	135 (6.2)	11 (4.9)	20 (5.8)	10 (5.7)	10 (3.2)	0.002*
Urinary tract infection	155 (2.9)	68 (3.4)	48 (2.2)	2 (0.9)	14 (4.1)	8 (4.6)	10 (3.2)	0.019*
Clinical symptoms, n (%)								
Any symptom	1715 (32.2)	762 (38.5)	626 (28.8)	31 (13.9)	113 (32.9)	70 (40)	65 (21.1)	< 0.001*
Pain	1211 (22.7)	568 (28.7)	414 (19.1)	21 (9.4)	79 (23)	52 (29.7)	38 (12.3)	< 0.001*
Gross hematuria	528 (9.9)	260 (13.1)	170 (7.8)	11 (4.9)	35 (10.2)	18 (10.3)	21 (6.8)	< 0.001*
Voiding symptoms	462 (8.7)	183 (9.2)	184 (8.5)	9 (4.0)	34 (9.9)	19 (10.9)	17 (5.5)	0.085
Urinary tract infection	218 (4.1)	102 (5.2)	65 (3.0)	2 (0.9)	19 (5.5)	10 (5.7)	13 (4.2)	0.001*
Acute retention of urine	154 (2.9)	64 (3.2)	55 (2.5)	1 (0.4)	12 (3.5)	10 (5.7)	6 (1.9)	0.023*
Asymptomatic	3616 (67.8)	1218 (61.5)	1544 (71.2)	192 (86.1)	230 (67.1)	105 (60.0)	243 (78.9)	< 0.001*

*PC* prostate cancer, *LPC/LAPC* localized PC/locally advanced PC, *RP* radical prostatectomy, *RT* radiotherapy, *BT* brachytherapy, *AS/WW* Active surveillance/Watchful waiting, *ADT* androgen deprivation therapy

Comorbidities were searched in the EHRs within a time window of (-Inf/0] around index date. Clinical symptoms were analyzed at index date with a window of (−3, 1] months. Cryotherapy and HIFU were not included as specific subgroups due to the low number of patients (5 and none, respectively) and were included in “Other”. Cases included in “Other” category are fully detailed in Table S3

Totals may not add up to the full cohort (*n* = 5331) as 132 patients with missing treatment classification were excluded from this table. The Chi-square test was used, except where indicated by (^&^), in which case the Kruskal–Wallis test was applied. ^*^Differences were considered statistically significant when *p* < 0.05. Adjustment of p-values for multiple comparisons was made using the Benjamini & Hochberg method

**Table 2:** Prostate cancer variables in LPC/LAPC patients at inclusion, overall and by initial treatment received

	**Selected LPC/LAPC** ***n* = 5,331**	**RP** ***n* = 1,980**	**RT** ***n* = 2,170**	**BT** ***n* = 223**	**AS/WW** ***n* = 343**	**ADT only** ***n* = 175**	**Other** ***n* = 308**	***p*-value**
PSA, ng/mL, median (Q1, Q3)	6.6 (4.5, 10.8)	5.6 (3.1, 8.6)	7.6 (5.0, 13.1)	6.3 (5.2, 9.2)	6.1 (4.5, 8.6)	13.4 (7.8, 29.2)	6.9 (4.8, 10.9)	< 0.001*^&^
Missing, n (%)	1769 (33.2)	669 (33.8)	705 (32.5)	72 (32.3)	116 (33.8)	62 (35.4)	85 (27.6)	
Rectal examination, n (%)*	2407 (45.2)	847 (42.8)	1008 (46.5)	86 (38.6)	170 (49.6)	85 (48.6)	161 (52.3)	0.013*
Suspicious	1248 (51.8)	387 (45.7)	589 (58.4)	51 (59.3)	58 (34.1)	58 (68.2)	76 (47.2)	< 0.001*
Undefined	920 (38.2)	338 (39.9)	368 (36.5)	25 (29.1)	82 (48.2)	28 (32.9)	61 (37.9)	0.003*
Normal	816 (33.9)	325 (38.4)	311 (30.9)	20 (23.3)	62 (36.5)	19 (22.4)	62 (38.5)	0.007*
Imaging procedures, n (%)*	3027 (56.8)	916 (46.3)	1424 (65.6)	159 (71.3)	169 (49.3)	107 (61.1)	173 (56.2)	< 0.001*
Prostate ultrasound	197 (6.5)	55 (6)	84 (5.9)	16 (10.1)	14 (8.3)	6 (5.6)	16 (9.2)	0.016*
CT (any location)	1343 (44.4)	441 (48.1)	643 (45.2)	35 (22)	54 (32)	56 (52.3)	74 (42.8)	< 0.001*
Bone scintigraphy	861 (28.4)	220 (24)	470 (33)	10 (6.3)	20 (11.8)	60 (56.1)	46 (26.6)	< 0.001*
MRI	1848 (61.1)	508 (55.5)	883 (62)	131 (82.4)	124 (73.4)	54 (50.5)	111 (64.2)	< 0.001*
MRI pelvic	121 (4.0)	42 (4.6)	63 (4.4)	2 (1.3)	4 (2.4)	4 (3.7)	3 (1.7)	0.125
MRI whole body	5 (0.2)	4 (0.4)	0 (0)	0 (0)	0 (0)	1 (0.9)	0 (0)	0.085
Positron emission tomography	175 (5.8)	52 (5.7)	92 (6.5)	2 (1.3)	4 (2.4)	8 (7.5)	5 (2.9)	0.001*
Prostate biopsy, n (%)	5227 (98.0)	1949 (98.4)	2132 (98.2)	216 (96.9)	326 (95.0)	170 (97.1)	303 (98.4)	0.001*
Complications, n (%)								
Post-biopsy hospital admission	651 (12.4)	252 (12.9)	300 (14.1)	18 (8.3)	22 (6.7)	31 (18.2)	18 (5.9)	< 0.001*
Gross hematuria	73 (1.4)	35 (1.8)	26 (1.2)	1 (0.5)	5 (1.5)	3 (1.8)	1 (0.3)	0.413
Infection	33 (0.6)	12 (0.6)	15 (0.7)	0 (0)	1 (0.3)	1 (0.6)	4 (1.3)	0.695
Gleason score, n (%)	5227 (98)	1949 (98.4)	2132 (98.2)	216 (96.9)	326 (95)	170 (97.1)	303 (98.4)	
≤ 6	2371 (44.5)	886 (44.7)	803 (37)	173 (77.6)	272 (79.3)	36 (20.6)	157 (51)	< 0.001*
7	2024 (38)	854 (43.1)	869 (40)	35 (15.7)	42 (12.2)	59 (33.7)	111 (36)	< 0.001*
≥ 8	832 (15.6)	209 (10.6)	460 (21.2)	8 (3.6)	12 (3.5)	75 (42.9)	35 (11.4)	< 0.001*
Without specific value reported	104 (2)	31 (1.6)	38 (1.8)	7 (3.1)	17 (5)	5 (2.9)	5 (1.6)	0.001*
Genomic testing, n (%)*	99 (1.9)	36 (1.8)	44 (2)	0 (0)	8 (2.3)	3 (1.7)	3 (1)	0.301
BRCA1 carriers	3 (3.0)	0 (0)	3 (6.8)	0 (0)	0 (0)	0 (0)	0 (0)	0.446
BRCA2 carriers	7 (7.1)	0 (0)	5 (11.4)	0 (0)	0 (0)	0 (0)	0 (0)	0.196
Risk categories, n (%)								
LR-LPC	391 (7.3)	87 (4.4)	130 (6)	53 (23.8)	77 (22.4)	1 (0.6)	34 (11)	< 0.001*
IR-LPC	1946 (36.5)	813 (41.1)	825 (38)	48 (21.5)	65 (19)	39 (22.3)	113 (36.7)	< 0.001*
HR-LPC	1386 (26.0)	427 (21.6)	701 (32.3)	34 (15.2)	30 (8.7)	78 (44.6)	71 (23.1)	< 0.001*
LAPC	317 (5.9)	86 (4.3)	142 (6.5)	6 (2.7)	0 (0)	40 (22.9)	25 (8.1)	< 0.001*
Unknown	1291 (24.2)	567 (28.6)	372 (17.1)	82 (36.8)	171 (49.9)	17 (9.7)	65 (21.1)	< 0.001*

*PC* prostate cancer, *LPC/LAPC* localized PC/locally advanced PC, *RP* radical prostatectomy, *RT* radiotherapy, *BT* brachytherapy, *AS/WW* Active surveillance/Watchful waiting, *ADT* androgen deprivation therapy, *PSA* Prostate-specific antigen, *CT* Computed tomography, *MRI* magnetic resonance imaging, *BRCA* breast cancer gene, *LR* low risk, *IR* intermediate risk, *HR* high risk

The presence of each feature for PSA and rectal examination was analyzed at inclusion with a window of (−3, 1] month and for imaging procedures and prostate biopsy at inclusion with a window of (−3, 3] months. Biopsy date was set for the nearest date to inclusion. The presence of each complication was searched with a window of (0/+ 15] days around the biopsy date. For Gleason the nearest value to the event date with no limit was taken. In case of conflict of two rectal examination results, both were included. However, in case of two conflict risk possible values for the same patient, the highest risk was assigned. Low risk: prostate specific antigen (PSA) < 10 ng/mL and cT1-T2a and Gleason score ≤ 7; intermediate risk: PSA 10–20 ng/mL or cT2b or Gleason score 7; high risk: PSA > 20 ng/mL or cT2c or Gleason score > 7; LAPC: cT3-4 or cN +; Unknown: non categorizable

^*^Percentages in these categories are calculated among tested patients

Cryotherapy and HIFU were not included as specific subgroups due to the low number of patients (5 and none, respectively) and were included in “Other”. Cases included in “Other” category are fully detailed in Table S3

Totals may not add up to the full cohort (*n* = 5331) as 132 patients with missing treatment classification were excluded from this table. The Chi-square test was used, except where indicated by (^&^), in which case the Kruskal–Wallis test was applied

^*^Differences were considered statistically significant when *p* < 0.05. Adjustment of p-values for multiple comparisons was made using the Benjamini & Hochberg method

Patients starting treatment with ADT only were the oldest (77 yr [69, 81]), had higher comorbidities rates (hypertension 56.0%, hypercholesterolemia 30.3%, and diabetes 25.1%), the highest median PSA levels (13.4 [7.8, 29.2] ng/mL), and the highest percentage of high-risk patients (44.6% HR-LPC and 22.9% LAPC). Patients starting treatment with RT were older than RP patients (71 yr [66, 75] and 66 yr [61, 70] respectively), and had more comorbidities (hypertension 45% and 37% and hypercholesterolemia 31% and 27%, respectively). Median PSA levels were higher for RT patients (7.6 [5.0, 13.1] ng/ml) compared to RP (5.6 [3.1, 8.6] ng/mL). A higher percentage of RT patients were in the high-risk groups (32.3% HR-LPC and 6.5% LAPC) compared to RP (21.6% HR-LPC and 4.3% LAPC).

### Health care resources utilization

Healthcare resource utilization was evaluated according to each patient’s initial treatment while they remained in the LPC/LAPC stage (Supplementary Table S6 and S7). Of selected patients analyzed, 92.5% attended at least one outpatient visit, with ADT-only and BT-treated patients having fewer visits (87.9% and 73.4% respectively). Additionally, 21.5% had at least one emergency visit, with notably lower rates among BT-treated patients (10.1%). Hospitalization was required for 40.5% of patients, with higher rates among BT- and RP- treated patients (77.8% and 55.9%, respectively).

### Outcomes

Estimated median (Q1, Q3) follow-up was 4.1 (4.1, 4.2) yr for the total LPC/LAPC cohort, and for patients selected for outcome analysis, it was 2.3 (1.0, 3.3) yr. In this last cohort, although the median rwOS and MFS was not reached, the median EFS was 33.7 months. Estimated median follow up, events and rwOS, EFS and MFS rates at 1, 2, 3 and 4 yr by risk group and by first treatment received subgroups are shown in Table 3. Median follow up was similar among subgroups. Distribution of follow up time among patients included in the outcome analysis is detailed in Table S8.

**Table 3:** Real world overal, event-free, and metastasis-free survival rates with follow-up medians for each subgroup analyzed

	**All**	**By risk**				**By first treatment**				
	**Selected LAPC/LPC**	**LAPC**	**HR-LPC**	**IR-LPC**	**LR-LPC**	**ADT only**	**AS/WW**	**BT**	**RP**	**RT**
Median Follow up (95% CI), years	2.25 (2.17–2.30)	2.11 (1.85–2.40)	2.30 (2.18–2.38)	2.25 (2.13–2.34)	2.01 (1.79–2.36)	2.53 (2.15–2.79)	2.08 (1.86–2.34)	2.11 (1.77–2.3)	2.45 (2.4–2.51)	1.98 (1.85–2.08)
Median Follow up (Q1-Q3), years	2.25 (1.02–3.30)	2.11 (0.95–3.66)	2.30 (1.11–3.39)	2.25 (1.00–3.13)	2.01 (0.90–3.39)	2.53 (1.40–3.48)	2.08 (1.00–3.31)	2.11 (1.02–3.05)	2.45 (1.31–3.45)	1.98 (0.81–3.17)
Real world overall Survival using oncology EHRs										
N	5,331	317	1,386	1,946	391	175	343	223	1,980	2,170
Deaths, n (%)	166 (3.1)	21 (6.6)	71 (5.1)	44 (2.3)	5 (1.3)	29 (16.6)	8 (2.3)	2 (0.9)	38 (1.9)	71 (3.3)
Median (95% CI)	NR	NR	NR	NR	NR	56.6 (54.2-NR)	NR	NR	NR	NR
1 year	99 (99–99)	97 (95–99)	99 (99–100)	99 (99–100)	100 (99–100)	97 (94–100)	100 (100–100)	100 (100–100)	99 (99–100)	99 (99–99)
2 years	98 (97–98)	93 (89–96)	97 (96–98)	98 (98–99)	99 (98–100)	89 (83–94)	99 (98–100)	99 (98–100)	99 (98–99)	98 (97–98)
3 years	96 (95–96)	91 (87–95)	93 (91–95)	97 (95–98)	98 (96–100)	79 (71–87)	97 (94–99)	98 (95–100)	98 (97–99)	95 (94–96)
4 years	94 (93–95)	91 (87–95)	90 (88–93)	95 (94–97)	96 (92–100)	73 (63–84)	93 (87–99)	98 (95–100)	97 (96–98)	92 (90–94)
Event Free Survival										
N	5,273	314	1,371	1,920	388	170	338	221	1,960	2,146
Events, n (%)	2,223 (42.2)	191 (60.8)	632 (46.1)	758 (39.5)	187 (48.2)	116 (68.2)	172 (50.9)	101 (45.7)	754 (38.5)	869 (40.5)
Median (95% CI)	33.7 (32.2–35.5)	17 (14.2–20.9)	30.8 (28.2–34)	35 (32.6–39.4)	28.5 (24.3–33.9)	15.6 (11.9–22.5)	25.8 (23.5–29.1)	29.8 (24.9–37.3)	41.7 (37.7–44.5)	33.4 (30.5–35.7)
1 year	79 (78–80)	59 (54–65)	76 (74–79)	81 (79–83)	76 (72–81)	55 (48–64)	76 (71–81)	80 (74–85)	82 (80–84)	80 (78–82)
2 years	62 (60–63)	42 (36–48)	58 (55–61)	64 (61–66)	56 (50–62)	38 (31–47)	54 (48–61)	60 (53–68)	67 (65–69)	62 (59–64)
3 years	48 (46–49)	32 (26–39)	42 (39–46)	49 (46–52)	41 (35–47)	26 (19–35)	35 (29–43)	43 (36–53)	55 (52–58)	47 (44–49)
4 years	34 (32–36)	23 (18–31)	31 (27–35)	35 (31–39)	27 (21–35)	15 (9–26)	20 (14–29)	12 (3–55)	43 (39–46)	32 (28–35)
Metastasis free Survival										
N	5,331	317	1,386	1,946	391	175	343	223	1,980	2,170
Metastasis, n (%)	329 (6.2)	67 (21.1)	136 (9.8)	74 (3.8)	12 (3.1)	59 (33.7)	9 (2.6)	10 (4.5)	88 (4.4)	93 (4.3)
Median (95% CI)	NR	NR	NR	NR	NR	NR	NR	NR	NR	NR
1 year	96 (95–996	84 (0.8–0.88)	93 (92–95)	98 (97–98)	99 (98–100)	77 (70–83)	98 (97–100)	97 (94–99)	97 (97–98)	98 (97–99)
2 years	94 (94–95)	79 (0.75–0.84)	91 (89–92)	96 (96–97)	97 (95–99)	67 (60–75)	97 (95–99)	96 (93–99)	96 (95–97)	96 (95–97)
3 years	92 (92–93)	77 (0.72–0.83)	87 (85–90)	95 (94–96)	96 (94–99)	61 (53–70)	96 (94–99)	96 (93–99)	95 (94–96)	94 (93–95)
4 years	90 (89–91)	71 (0.65–0.78)	86 (84–89)	94 (92–95)	92 (86–98)	56 (47–67)	96 (94–99)	91 (85–99)	93 (91–95)	92 (90–94)

*PC* prostate cancer, *LPC/LAPC* localized PC/locally advanced PC, *RP* radical prostatectomy, *RT* radiotherapy, *BT* brachytherapy, *AS/WW* Active surveillance/Watchful waiting, *ADT* androgen deprivation therapy

**Fig. 3:**
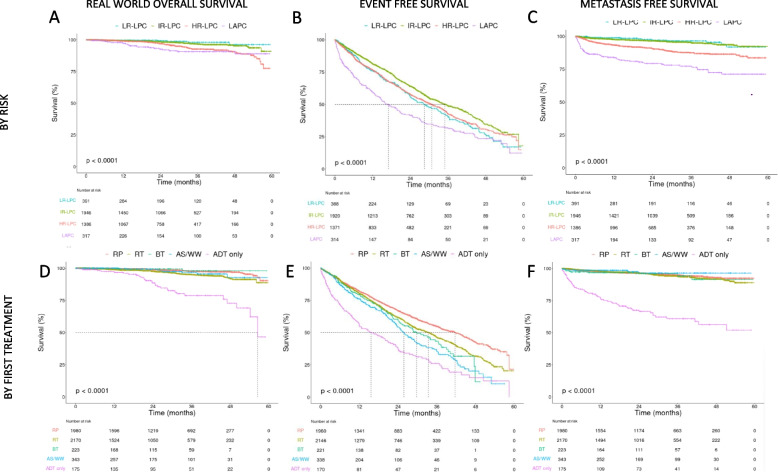
Outcomes. Real world overall survival using oncology EHRs (left panels, A and D), Event free survival (middle panels, B and E) and metastasis free survival (right panels, C and F) analysis using Kaplan–Meier method, for patients with Prostate cancer (PC) stratified by risk (first row, A, B and C) and by first treatment received (second row, D, E and F). Note: Kaplan–Meier survival comparisons shown are unadjusted and intended for descriptive purposes only. PC: prostate cancer; LPC/LAPC: localized PC/locally advanced PC; LR: low risk; IR: intermediate risk; HR: high risk; Unk risk: unknown risk; RP: radical prostatectomy; RT: radiotherapy; BT: brachytherapy; AS/WW: Active surveillance/Watchful waiting; ADT: androgen deprivation therapy

Outcomes for patients stratified by D’Amico risk groups showed that clinical outcomes worsened as LPC risk increased. rwOS at 36 months was 91% for LAPC patients, 93% for HR-LPC, 97% for IR-LPC and 98% for LR-LPC (Fig. 3A and Table 3). EFS at 36 months were 32% for LAPC patients, 42% for HR-LPC, 49% for IR-LPC and 41% for LR-LPC; and median time to event was 17, 30.8, 35 and 28.5 months respectively (Fig. 3B and Table 3). Worse EFS for LR compared to IR were primarily driven by PSA failure events (Supplementary Figure S2). MFS at 36 months was 77% for LAPC patients, 87% for HR-LPC, 95% for IR-LPC and 96% for LR-LPC (Fig. 3C and Table 3).

Clinical outcomes based on the first treatment received were analyzed. Of note, the treatment groups were not well balanced in terms of baseline characteristics or disease severity. Median follow-up for all treatment groups ranged from 1.98 to 2.53 years (Table 3). At 36 months, rwOS varied, with the lower percentages in the ADT only group (79%) and the highest rates for BT and RP (both 98%) (Fig. 3D and Table 3). For EFS at 36 months, the RP group had the highest percentage, with 55% of patients without an event. The other treatment groups had lower values: ADT only (26%), AS/WW (35%), BT (43%), and RT (47%). Median time to event (95% CI) were 41.7 months (37.7–44.5) for RP-treated patients; 33.4 months (30.5–35.7) for RT-treated patients; 29.8 months (24.9–37.3) for BT-treated patients; 25.8 months (23.5–29.1) for AS/WW; and 15.6 months (11.9–22.5) for ADT only treated patients (Fig. 3E and Table 3). The percentage of patients developing metastases during follow up was highest for patients treated with ADT only (33.7%) and similar among the other treatment groups (2.6%−4.5%) (Fig. 3F and Table 3).

## Discussion

In this study, AI was used to extract information from EHRs of PC patients treated in eight Spanish hospitals. 22,166 PC patients were identified from a population of 2,626,612 patients seen at the participating hospitals during the study period. The proportion of patients identified as LPC/LAPC (65%) was slightly lower than previously reported estimates (~ 80%), which may reflect real-world limitations including incomplete staging information, the underrepresentation of low-risk patients often managed in primary care or under active surveillance, and the fact that the eight participating hospitals are tertiary centers, likely receiving a higher proportion of referred patients with advanced disease.

We found an annual incidence rate for LPC/LAPC of 91.9 per 100,000 persons per year in 2018, which is consistent with the 2022 crude rate of new cases of PC in Spain of 116.1 per 100,000 [26]. *BRCA* testing was recorded in a very small percentage of patients, but a progressive increase in genetic testing may be expected. Intermediate risk (IR)-LPC was the most frequent group at inclusion. Additionally, a high percentage of patients required outpatient visits (92.5%), while around 20% and 40% needed emergency care and hospitalization, respectively, reflecting the healthcare burden these patients represent.

During a median follow-up period of 2.3 yr, the mortality rate was 3.1% among all patients. However, a lower rwOS at 36 months was observed among patients with LAPC (91%) and HR-LPC (93%) groups. MFS at 36 months was 92% for all patients and lower in LAPC patients (77%) and in HR-LPC patients (87%), despite the fact that the majority of these patients were treated with RT or RP. The outcomes stratified by risk groups revealed a clear trend of worsening prognosis with increasing PC risk and highlights the need for early identification and treatment with curative-intent such as RP and RT for patients classified as HR-LPC or LAPC. This aligns with previous studies that have demonstrated poorer outcomes in higher-risk PC groups [27].

The proportions of patients receiving each evaluated treatment across risk categories in our study are consistent with those documented in previous studies [5]. In our cohort of LPC/LAPC patients, RT and RP were the preferred first line treatments used in 40.7% and 37.1% of cases, respectively. Similar to other reports, patients initially receiving RT were older, had more comorbidities, and higher PSA, than patients treated with RP [28–30]. Patients treated with ADT only showed worse baseline characteristics compared to other groups, particularly in terms of age, cancer stage, and comorbidities, which likely influenced treatment selection and may have also contributed to poorer outcomes in terms of rwOS, EFS and MFS. In our analysis, this group included only patients who had no record of curative local therapies (e.g., RP, RT, BT) at treatment initiation nor during the follow-up period while classified as LPC/LAPC. Therefore, this subgroup likely represents patients for whom curative treatment was either not indicated, not feasible, or not pursued—due to clinical judgment, comorbidities, or patient preference. These findings are consistent with existing literature suggesting ADT only may be less effective compared to other treatment options [31].

Randomized trials comparing the efficacy of RT vs RP in LPC are currently lacking. Our study showed similar outcomes for rwOS and MFS and differences in favour of RP for EFS, however these results should be interpreted cautiously as the two cohorts were not balanced and RT patients presented with worse baseline prognostic characteristics. In a systematic review of administrative and registry studies with a pooled population of 228,444 patients, RT was associated with worse overall and cancer-specific mortality than RP, and any active treatment with better outcome as compared to no treatment, observation, or AS [31]. Accordingly, the election between RP and RT should be made on an individual basis, considering patient-specific factors such as age, comorbidities, and personal preferences. In accordance with previous reports, our findings suggest that, due to its risk of relapse, treatment with curative intent is essential for both HR-LPC and LAPC patients [32]. In addition, our results also suggest that ADT only is insufficient to achieve favorable outcomes.

This multicenter study leveraged primarily free-text data from EHRs, supplemented by structured data when available, using NLP and ML to extract clinical information on LPC/LAPC patients in real-world settings. This approach enabled richer and more consistent data capture across hospitals with heterogeneous EHR systems, overcoming the limitations of non-uniform coding practices—particularly relevant in settings like Spain. Beyond characterizing management and outcomes of LPC/LAPC, the study highlights the potential of AI-driven methods to support large-scale real-world data generation and lays the groundwork for future registry development, clinical monitoring, and healthcare system planning.

This study has several limitations inherent to real-world EHR data. First, the quality of results depends on the completeness and consistency of clinical documentation. Some relevant variables (e.g., radiotherapy dose, ADT duration) could not be extracted due to heterogeneous or incomplete documentation. Additionally, missing parameters such as PSA or Gleason score, led to the exclusion of some patients from outcome analyses to ensure reliable risk stratification. However, the majority of excluded patients were non-incident cases, which could otherwise have introduced bias into outcome estimates. Second, the extraction performance of certain outcome-related terms (e.g., metastatic PC, PSA response) was lower, potentially affecting subgroup definitions. However, high performance in broader concepts like ‘prostate cancer’ and general ‘metastasis’ may have mitigated this impact. Third, although p-values were included to support interpretation, the study was not designed for comparative analysis. No multivariable adjustments were made, and findings should be interpreted as descriptive. Fourth, the median follow-up of 2.3 years limits long-term outcome interpretation, particularly for a slow-progressing disease like PC. Still, most patients had over one year of follow-up, providing a meaningful view of short- to mid-term outcomes. Finally, the 2014–2018 study period reflects care prior to the adoption of PSMA-PET and other innovations. Nonetheless, it offers a valuable real-world snapshot of established clinical practice. The study also highlights the feasibility of AI-driven data extraction at scale, especially in settings where structured data are limited and unstructured text is key.

## Conclusions

The OVERVIEW study defined the clinical profile of patients with LPC/LAPC using AI for data extraction from EHRs. It included 5,331 LPC/LAPC patients with a median follow-up of 2.3 years. RT and RP were the most common initial treatments. Patients on ADT monotherapy showed poorer clinical outcomes, likely influenced by both the suboptimal nature of the treatment and unfavorable baseline characteristics. The study highlights the need to optimize treatment with curative intent in LAPC/HR-LPC subgroups, as a significant proportion of patients (1–2 out of 10) still develop metastases despite receiving such therapy. A better understanding of the LPC/LAPC landscape is crucial for optimizing management and outcomes through personalized treatment strategies. Additionally, the study demonstrates the feasibility of conducting RWE studies using AI, producing results consistent with previous data.

## Supplementary Information

Supplementary Material 1.

## Data Availability

The original contributions presented in the study are included in the article/supplementary material, further inquiries can be directed to the corresponding author.

## Abbreviations

ADT: Androgen deprivation therapy
AI: Artificial intelligence
AS: Active surveillance
BT: Brachytherapy
EAU: European Association of Urology
EFS: Event free survival
EHR: Electronic health records
GS: Gleason score
HR: High risk
IR: Intermediate risk
LAPC: Locally advanced prostate cancer
LPC: Localized prostate cancer
LR: Low risk
NLP: Natural language processing
MFS: Metastasis free survival
ML: Machine learning
MRI: Magnetic resonance imaging
PC: Prostate cancer
PSA: Prostate specific antigen
RP: Radical prostatectomy
RT: Radiotherapy
RWD: Real-world data
RWE: Real-world evidence
rwOS: Real world overall survival using oncology EHRs
SNOMED CT: Systematized Nomenclature of Medicine – Clinical Terms
WW: Watchful waiting
yr: Year
