# Supplementary material for: Real-world evidence in localized and locally advanced prostate cancer: applying artificial intelligence to electronic health records
Source: BMC Cancer. 2025 Oct 21;25:1618. doi: 10.1186/s12885-025-14828-z (PMC12539219; doi:10.1186/s12885-025-14828-z)
Supplement: Supplementary file 1 — Supplementary Material 1. [file 12885_2025_14828_MOESM1_ESM.docx]

**Real-World Evidence in Localized and Locally Advanced Prostate Cancer Applying Artificial Intelligence to Electronic Health Records**

**SUPPLEMENTAL MATERIALS**

Table of Contents

[Supplemental methods 3](#_Toc203472693)

[Study design and population 3](#_Toc203472694)

[Stratifications 3](#_Toc203472695)

[Extraction of the Unstructured Information from Electronic Health Records 4](#_Toc203472696)

[EHRead® performance 4](#_Toc203472697)

[Outcomes 6](#_Toc203472698)

[Study variables 7](#_Toc203472699)

[Acknowledgements 7](#_Toc203472700)

[Supplemental results 9](#_Toc203472701)

[References 20](#_Toc203472702)

**Supplemental Tables**

[Supplemental Table 1. Participating centers by region. 9](#_Toc203472703)

[Supplemental Table 2. Reading performance of EHRead® Technology. 10](#_Toc203472704)

[Supplemental Table 3. Demographic and Clinical Characteristics of Patients with Localized and Locally Advanced Prostate Cancer (LPC/LAPC) at inclusion. 11](#_Toc203472705)

[Supplemental Table 4. Variables related with Prostate Cancer in Patients with Localized and Locally Advanced (LPC/LAPC) disease at inclusion. 12](#_Toc203472706)

[Supplemental Table 5. Patients included in the “Other” group of the First treatment with curative intent stratification. 14](#_Toc203472707)

[Supplemental Table 6. Healthcare Resource Utilization Related to LPC/LAPC patients during localized stage, overall and by first treatment received. 15](#_Toc203472708)

[Supplemental Table 7. Healthcare Resource Utilization Related to Prostate Cancer During Localized Stage. 16](#_Toc203472709)

[Supplemental Table 8. Distribution of follow up time among patients included in the outcome analysis. 17](#_Toc203472710)

**Supplemental Figures**

[Supplemental Figure 1. UpSet plot of applied filters and resulting patient subsets 18](#_Toc203472711)

[Supplemental Figure 2. Event free survival using Kaplan-Meier method for patients with prostate cancer 19](#_Toc203472712)

# Supplemental methods

## Study design and population

To classify patients as having LPC or LAPC, we selected individuals from the OVERVIEW study who, at any point in their disease trajectory, met at least one of the following criteria: i) Diagnosis of PC with TNM staging indicating M0; for LAPC: T3, T4, or N1; for LPC: T1 or T2 with N0); ii) Diagnosis of PC with corresponding clinical stage: for LPC stages I, II, IIA, IIB, III, or IIIA; for LAPC stages IIIB, IIIC, or IVA; iii) Explicit mention of “LPC” (or their synonyms or acronyms) in clinical terminology or iv) Explicit mention of “LAPC” (or their synonyms or acronyms) in clinical terminology.

Follow up period was censored at the end of the study period and loss of follow-up was determined by specific criteria, including: > 1 month or > 3 months from hospital referral to palliative care or to home monitoring, respectively; > 3 months from discharge with an Eastern Cooperative Oncology Group (ECOG) status ≥ 3; > 6 months for patients who had progressed to mCRPC or terminal stage as documented in the EHR.

## Stratifications

Patients were stratified into risk groups based on terminology explicitly mentioned in the clinical text or, when not available, based on calculated risk using the D’Amico classification [1], applying the following criteria:

- Low risk LPC (LR-LPC): PSA < 10 ng/mL and clinical stage T1–T2a and Gleason score ≤ 7
- Intermediate risk LPC (IR-LPC): PSA 10–20 ng/mL or clinical stage T2b or Gleason score = 7
- High risk LPC (HR-LPC): PSA > 20 ng/mL or clinical stage T2c or higher or Gleason score > 7
- LAPC: cT3-4 or cN+
- Unknown risk: Patients with missing or incomplete data (e.g., missing PSA, Gleason score, and/or TNM staging) who could not be classified based on the criteria above.

## Extraction of the Unstructured Information from Electronic Health Records

All study variables were extracted from patients Electronic Health Records (EHRs) using EHRead® technology which uses Natural language processing (NLP) and Machine learning (ML) techniques for extracting clinical information from EHRs which is translated into concepts, synonyms, and definitions using specific terminology based on Systematized Nomenclature of Medicine—Clinical Terms (SNOMED-CT). Subsequently, the information is systematically organized and converted into a structured synthetic study database. This process required that conceptual definitions for all study variables were pre-specified and aligned with clinical entities found in the SNOMED Clinical Terms (a comprehensive, computationally processable collection of medical terms utilized in clinical documentation) using the SNOMED CT browser. This step facilitated the conversion of unstructured data from various hospital departments into actionable variables for extraction. The clinical accuracy of the conceptual definitions and entity mapping was reviewed and approved by medical research experts specialized in NLP.

Once the clinical entities were extracted, variables were constructed by applying dedicated data wrangling operations to their mapped entities, leveraging specific NLP parameters generated by dedicated ML models (e.g., negation, temporality, attributes, etc.) and record-specific metadata (e.g., date, medical department, record type, etc.).

## EHRead® performance

To ensure the quality of data extraction, the performance of EHRead® was externally evaluated. Specifically, this validation was carried out by external annotators following a peer-reviewed method [2]. The evaluation of the performance of *EHRead^®^* involved the following phases:

- *Text collection*. In NLP systems, the amount of data necessary to capture enough linguistic events to ensure consistent and robust performance metrics is an open question. To address this, we utilized the Sample Calculator for Evaluation (SLiCE®), a software tool specifically developed for this purpose. This calculator indicates the minimum number of annotated EHRs required to obtain the expected parameters based on the prevalence in the EHRs of the main study variable. The parameters used for this calculation included a confidence level of 95% (α = 5%), interval widths of 10% (percentage points) and expected values of precision and recall. Thus, SliCE provides a robust estimation of precision and recall assuring that the true value is at ±5% (percentage points) with a confidence level of 95%.
- *Annotation task*. The overall goal of this phase was to evaluate the system’s accuracy when identifying records that contained mentions of PC-related variables. To build the standard corpus, a set of documents was first pre-annotated using *EHRead^®^* technology; these documents included key study variables to identify the population with PC. Then, these documents were corrected manually via in-house developed Evaluation Tool.
- *Annotation of the standard*. Two designated expert physicians (hereby referred to as ‘the annotators’) at each hospital annotated the set of randomly selected records. Annotators adhered to the annotation guidelines developed by the medical team of NLP experts. Then, the Inter-Annotator Agreement (IAA) was measured using the F1-Score to ensure the consistency of the guidelines and the reliability of the annotation. The IAA is a metric that indicates the extent to which the different annotators converged in their evaluation, thus providing information regarding the difficulty of the task. Finally, a third physician acted as judge, reviewing the annotations made by the two annotators and resolving any possible discrepancies. The resulting standard corpus served as a resource for the evaluation of the performance of EHRead® technology.
- *Evaluation*. The evaluation of the system was calculated in terms of the standard metrics of Precision, Recall, and their harmonic mean F1-Score.
- *Precision* =
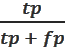
. This parameter indicates the accuracy of the system in retrieving key clinical concepts.
- *Recall* =
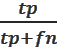
 . This parameter indicates the amount of information the system retrieves.
- *F1-Score* =
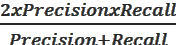
. This parameter gives us an overall performance indicator of information retrieval.

In all cases, *tp* is the number of true positives (i.e., records correctly retrieved), *fn* is the set of false negatives (i.e., records incorrectly not retrieved), and *fp* is the number of false positives (i.e., records incorrectly retrieved).

The results of EHRead® performance metrics for this study are shown in **Table S2**.

## Outcomes

For real world overall survival using oncology EHRs (rwOS), death was inferred if any of the following occurred: time from discharge to palliative care in hospital of >1 month, time from discharge to palliative care at home of >3 months, time from discharge with an ECOG ≥3 of >3 months, and, for those patients that progress until mCRPC stage or "terminal stage" detected in the EHR, loss of follow-up > 6 months.

For EFS an event was considered if any of the following circumstances occurred: PSA failure (defined as a rise ≥2ng/ml above PSA nadir after treatment), local or regional recurrence, metastatic progression (as previously described), transition to another stage (biochemical recurrence, non-metastatic castration-resistant PC, metastatic hormone-sensitive PC, metastatic castration-resistant PC), or death.

## Study variables

Study variables related to demographic characteristics (such as age and sex), laboratory values and resource utilization variables, such as department visits and hospitalizations, were extracted from structured text. Visits/100 patients per year were calculated as the sum of all observed uses of each resource divided by the sum of all observed follow-up times, multiplied by 100. For the assessment of hospital admission, only patients with at least one hospital admission were evaluated. Variables related to comorbidities, clinicopathological characteristics, treatment, and disease outcomes were extracted from unstructured text. Free-text variables underwent a selection and curation process done by medical research experts in NLP that sought to guarantee their quality and integrity. To reconstruct patient background all information before index date was analyzed. For variables analyzed around discrete time points, the closest value to the time point within reference time windows was taken. Reference time windows accounted for the variability in healthcare management between patients, specialists, and hospitals, maximizing data retrieval from EHRs. Time window ranges for each variable or group of variables are detailed in table footnotes.

## Acknowledgements

The authors wish to thank the investigators who participated in the OVERVIEW study: Verónica Rodríguez Teesedo, Coral Manso, Cristina Beatriz Gutiérrez Ruiz, y Manuel A. Ruiz Serrano (Hospital Río Hortega, Valladolid); Angel Rodriguez, Miguel Ángel Alonso, y José Reyes Rodriguez Garrido (Hospital de León, León); Gemma Sancho, Jose Pablo Maroto, y Georgia Anguera Palacios (Hospital Santa Creu i Sant Pau, Barcelona); Joan Carles Galcerán, Xavier Maldonado, y Juan Morote (Hospital Vall d'Hebron, Barcelona); Virginia Hernández, Ana Guijarro, y Carlos Llorente (Hospital Universitario de Alcorcón, Alcorcón); Victor Carrero y Almudena Martín (Hospital Infanta Leonor, Madrid); Nuria Romero y Carlos Olivier (Hospital La Princesa, Madrid); Antonio Conde y César David Vera-Donoso (Hospital Universitario La Fe, Valencia); Jose Manuel de la Morena, Jorge Caño, y Erika Mateo (Hospital Infanta Sofía, Madrid); Begoña Caballero, Silva, y Álvaro Páez (Hospital de Fuenlabrada, Fuenlabrada); Josefa Terrasa, Enrique Pieras, y Jose Pardo (Hospital Son Espases, Palma de Mallorca); e Isabel Chirivella, Jose María Martínez Jabaloyas, Eduardo Ferrer, y Raquel García (Hospital Universitario Clínico de Valencia, Valencia).

# Supplemental results

Supplemental Table 1. Participating centers by region.

| **Center** | **Region** |
| --- | --- |
| Hospital Universitario de Fuenlabrada | Madrid |
| Hospital Universitario Fundación Alcorcón | Madrid |
| Hospital Universitario Infanta Leonor | Madrid |
| Hospital Universitario Infanta Sofía | Madrid |
| Hospital Clínico Universitario de Valencia | Valencia |
| Hospital Universitario y Politécnico La Fe | Valencia |
| Hospital Universitario Vall d´Hebron | Catalonia |
| Hospital Universitario Son Espases | Balearic Islands |

Supplemental Table 2. Reading performance of EHRead® Technology.

| **Variable** | **TP** | **FP** | **FN** | **Recall** | **Precision** | **F1 Score** | **Agreement**  **(F1 Score)** |
| --- | --- | --- | --- | --- | --- | --- | --- |
| PSA | 5018 | 1059 | 327 | 0.939 | 0.826 | 0.879 | 0.944 |
| Hematuria | 4857 | 1223 | 320 | 0.938 | 0.799 | 0.863 | 0.980 |
| Radiotherapy | 3301 | 716 | 627 | 0.840 | 0.822 | 0.831 | 0.928 |
| Biopsy | 2508 | 496 | 192 | 0.929 | 0.835 | 0.879 | 0.989 |
| Metastasis | 2520 | 425 | 231 | 0.916 | 0.856 | 0.885 | 0.952 |
| Systemic arterial hypertensive disorder | 1781 | 281 | 113 | 0.940 | 0.864 | 0.900 | 0.981 |
| Gleason | 1561 | 198 | 140 | 0.918 | 0.887 | 0.902 | 0.988 |
| Hormonal blockade | 1213 | 219 | 170 | 0.877 | 0.847 | 0.862 | 0.921 |
| Radical prostatectomy | 1198 | 93 | 193 | 0.861 | 0.928 | 0.893 | 0.832 |
| Fall | 842 | 190 | 449 | 0.652 | 0.816 | 0.725 | 0.854 |
| Docetaxel | 591 | 85 | 7 | 0.988 | 0.874 | 0.928 | 0.987 |
| Prostate cancer | 3020 | 284 | 794 | 0.792 | 0.914 | 0.849 | 0.948 |
| Biochemical recurrence | 582 | 33 | 275 | 0.679 | 0.946 | 0.791 | 0.920 |
| Urine infection | 507 | 22 | 132 | 0.793 | 0.958 | 0.868 | 0.931 |
| Abiraterone | 317 | 50 | 21 | 0.938 | 0.864 | 0.899 | 0.947 |
| Enzalutamide | 337 | 20 | 13 | 0.963 | 0.944 | 0.953 | 0.987 |
| Cabazitaxel | 211 | 20 | 51 | 0.805 | 0.913 | 0.856 | 0.958 |
| PSA Response | 216 | 3 | 125 | 0.633 | 0.986 | 0.771 | 0.924 |
| Brachytherapy | 137 | 37 | 10 | 0.932 | 0.787 | 0.854 | 0.946 |
| Radio 223 | 65 | 7 | 11 | 0.855 | 0.903 | 0.878 | 0.920 |
| Orchiectomy | 28 | 9 | 3 | 0.903 | 0.757 | 0.824 | 1.000 |
| Prostate ultrasound | 17 | 10 | 4 | 0.810 | 0.630 | 0.708 | 0.791 |
| Metastatic prostate cancer | 22 | 3 | 89 | 0.198 | 0.880 | 0.324 | 0.381 |
| Apalutamide | 8 | 1 | 0 | 1.000 | 0.889 | 0.941 | 0.933 |
| Metastatic hormone-sensitive prostate cancer | 3 | 0 | 1 | 0.750 | 1.000 | 0.857 | 0.857 |

PSA: Prostate-specific antigen; TP: true positives; FP: false positives; FN: false negatives.

Supplemental Table 3. Demographic and Clinical Characteristics of Patients with Localized and Locally Advanced Prostate Cancer (LPC/LAPC) at inclusion.

|  | **Selected LPC/LAPC**  **n =5,331** | **Non-Selected LPC/LAPC**  **n=9,103** | **p-value** | **Total LPC/LAPC**  **n = 14,434** |
| --- | --- | --- | --- | --- |
| **Age, years, median (Q1, Q3)** | 68 (63, 73) | 69 (63, 75) | 0.003*^&^ | 69 (63, 74) |
| **Age groups n (%)** |  |  |  |  |
| < 65 years | 1668 (31.3) | 2852 (31.3) | 0.970 | 4,520 (31.3) |
| 65-75 years | 2783 (52.2) | 4295 (47.2) | <0.001* | 7,078 (49.0) |
| > 75 years | 880 (16.5) | 1956 (21.5) | <0.001* | 2,836 (19.6) |
| **Family history of PCa, n (%)** | 775 (14.5) | 944 (10.4) | <0.001* | 1,719 (11.9) |
| **Current smokers, n (%)** | 968 (18.2) | 2103 (23.1) | <0.001* | 3,071 (21.3) |
| **Alcohol use, n (%)** | 399 (7.5) | 727 (8.0) | 0.321 | 1,126 (7.8) |
| **Main comorbidities, n (%)** |  |  |  |  |
| Hypertension | 2219 (41.6) | 2674 (29.4) | <0.001* | 4,893 (33.9) |
| Hypercholesterolemia | 1505 (28.2) | 1597 (17.5) | <0.001* | 3,102 (21.5) |
| Diabetes mellitus | 917 (17.2) | 1196 (13.1) | <0.001* | 2,113 (14.6) |
| Chronic obstructive pulmonary disease | 501 (9.4) | 423 (4.6) | <0.001* | 997 (6.9) |
| Chronic liver dysfunction | 313 (5.9) | 571 (6.3) | 0.375 | 924 (6.4) |
| Cerebrovascular event | 261 (4.9) | 528 (5.8) | 0.026* | 884 (6.1) |
| Myocardial infarction | 295 (5.5) | 486 (5.3) | 0.642 | 789 (5.5) |
| Chronic kidney disease | 372 (7) | 257 (2.8) | <0.001* | 781 (5.4) |
| Obstructive sleep apnea | 155 (2.9) | 175 (1.9) | <0.001* | 629 (4.4) |
| Urinary tract infection | 2219 (41.6) | 2674 (29.4) | <0.001* | 330 (2.3) |
| **Clinical symptoms, n (%)** |  |  |  |  |
| Any symptom | 1715 (32.2) | 1977 (21.7) | <0.001* | 3,692 (25.6) |
| Pain | 1211 (22.7) | 1594 (17.5) | <0.001* | 2,805 (19.4) |
| Gross hematuria | 528 (9.9) | 550 (6) | <0.001* | 1,078 (7.5) |
| Voiding symptoms | 462 (8.7) | 287 (3.2) | <0.001* | 749 (5.2) |
| Urinary tract infection | 218 (4.1) | 234 (2.6) | <0.001* | 452 (3.1) |
| Acute retention of urine | 154 (2.9) | 177 (1.9) | <0.001* | 331 (2.3) |
| Asymptomatic | 3616 (67.8) | 7126 (78.3) | <0.001* | 10,742 (74.4) |

Comorbidities were searched in the EHRs within a time window of (-Inf/0] around index date. Clinical symptoms were analyzed at index date with a window of (-3, 1] months. PC: prostate cancer; LPC/LAPC: localized PC/locally advanced PC. Fisher's Exact Test for Count Data (two.sided) was used except for ^&^ where Welch Two Sample t-test (two.sided) was used. * Differences were considered statistically significant when p < 0.05.

Supplemental Table 4. Variables related with Prostate Cancer in Patients with Localized and Locally Advanced (LPC/LAPC) disease at inclusion.

|  | **Selected LPC/LAPC**  **n=5331** | **Non-Selected LPC/LAPC**  **n=9103** | **p-value** | **Total LPC/LAPC**  **n = 14,434** |
| --- | --- | --- | --- | --- |
| **Prostate-specific antigen (PSA), ng/mL, median (Q1, Q3)** | 6.6 (4.5, 10.8) | 5.6 (0.5, 10.0) | 0.011*^&^ | 6.1 (2.6-10.5) |
| **Rectal examination, n (%)*** | 2407 (45.2) | 1495 (16.4) | <0.001* | 3,902 (27.0) |
| Suspicious | 1248 (51.8) | 739 (49.4) | <0.001* | 1,987 (50.9) |
| Undefined | 920 (38.2) | 634 (42.4) | <0.001* | 1,554 (39.8) |
| Normal | 816 (33.9) | 543 (36.3) | <0.001* | 1,359 (34.8) |
| **Imaging procedures, n (%)*** | 3027 (56.8) | 2284 (25.1) | <0.001* | 5,311 (36.8) |
| Prostate ultrasound | 197 (6.5) | 157 (6.9) | <0.001* | 354 (6.7) |
| Computed tomography (CT) (any location) | 1343 (44.4) | 1167 (51.1) | <0.001* | 2,510 (47.3) |
| Bone scintigraphy | 861 (28.4) | 630 (27.6) | <0.001* | 1,491 (28.1) |
| Magnetic resonance imaging (MRI) | 1848 (61.1) | 1285 (56.3) | <0.001* | 3,133 (59.0) |
| MRI pelvic | 121 (4.0) | 66 (2.9) | <0.001* | 187 (3.5) |
| MRI whole body | 5 (0.2) | 1 (0) | 0.032* | 6 (0.1) |
| Positron emission tomography (PET) | 175 (5.8) | 165 (7.2) | <0.001* | 340 (6.4) |
| **Prostate biopsy, n (%)** | 5227 (98.0) | 5236 (57.5) | <0.001* | 10,463 (72.5) |
| **Complications, n (%)** |  |  |  |  |
| Post-biopsy hospital admission | 651 (12.5) | 337 (6.4) | <0.001* | 988 (9.4) |
| Gross hematuria | 73 (1.4) | 29 (0.6) | <0.001* | 102 (1.0) |
| Infection | 33 (0.6) | 17 (0.3) | <0.001* | 50 (0.5) |
| **Gleason score, n (%)** |  |  |  |  |
| ≤ 6 | 2371 (44.5) | 2387 (26.2) | <0.001* | 4,758 (33.0) |
| 7 | 2024 (38) | 1881 (20.7) | <0.001* | 3,905 (27.1) |
| ≥ 8 | 832 (15.6) | 968 (10.6) | <0.001* | 1,800 (12.5) |
| Missing | 104 (2) | 3867 (42.5) | <0.001* | 3,971 (27.5) |
| **Genomic testing, n (%)*** | **99 (1.9)** | 225 (2.5) | 0.020* | 324 (2.2) |
| BRCA1 carriers | 3 (3.0) | 10 (4.4) | 0.417 | 13 (4.0) |
| BRCA2 carriers | 7 (7.1) | 15 (6.7) | 0.678 | 22 (6.8) |
| **Risk categories, n (%)** |  |  |  |  |
| LR-LPC | 391 (7.3) | 427 (4.7) | <0.001* | 818 (5.7) |
| IR-LPC | 1946 (36.5) | 1822 (20) | <0.001* | 3,768 (26.1) |
| HR-LPC | 1386 (26) | 1558 (17.1) | <0.001* | 2,944 (20.4) |
| LAPC | 317 (5.9) | 579 (6.4) | 0.366 | 896 (6.2) |
| Unknown | 1291 (24.2) | 4717 (51.8) | <0.001* | 6,008 (41.6) |

The presence of each feature for PSA and rectal examination was analyzed at inclusion with a window of (-3, 1] month and for imaging procedures and prostate biopsy at inclusion with a window of (-3, 3] months. Biopsy date was set for the nearest date to inclusion. The presence of each complication was searched with a window of (0/+ 15] days around the biopsy date. For Gleason the nearest value to the event date with no limit was taken. In case of conflict of two rectal examination results, both were included. However, in case of two conflict risk possible values for the same patient, the highest risk was assigned. Low risk: prostate specific antigen (PSA) < 10 ng/mL and cT1-T2a and Gleason score ≤ 7; intermediate risk: PSA 10-20 ng/mL or cT2b or Gleason score 7; high risk: PSA > 20 ng/mL or cT2c or Gleason score > 7; LAPC: cT3-4 or cN+; Unknown: non categorizable. PC: prostate cancer; LPC/LAPC: localized PC/locally advanced PC.

* Percentages in these categories are calculated among tested patients.

Fisher's Exact Test for Count Data (two.sided) was used except for ^&^ where Welch Two Sample t-test (two.sided) was used. * Differences were considered statistically significant when p < 0.05.

Supplemental Table 5. Patients included in the “Other” group of the First treatment with curative intent stratification.

| **n (%)** | **Selected LPC/LAPC**  **n = 5,331** |
| --- | --- |
| “Other” group | 308 (5.8) |
| Radical prostatectomy-Radiotherapy | 135 (2.5) |
| Brachytherapy-Radiotherapy | 121 (2.3) |
| Brachytherapy-Cryotherapy-Radical prostatectomy-Radiotherapy | 19 (0.4) |
| Brachytherapy-Cryotherapy-Radiotherapy | 12 (0.2) |
| Brachytherapy -Radical prostatectomy-Radiotherapy | 9 (0.2) |
| Brachytherapy -Radical prostatectomy | 6 (0.1) |
| Cryotherapy | 5 (0.1) |
| Cryotherapy-Radical prostatectomy | 1 (0.02) |

PC: prostate cancer; LPC/LAPC: localized PC/locally advanced PC

Supplemental Table 6. Healthcare Resource Utilization Related to LPC/LAPC patients during localized stage, overall and by first treatment received.

|  | | **Selected LPC/LAPC**  **n = 5,331** | | **RP**  **n = 1,980** | | **RT**  **n = 2,170** | | **BT**  **n = 223** | | **AS/WW**  **n = 343** | | **ADT only**  **n = 175** | | **Other**  **n = 308** | | **p-value** | |
| --- | --- | --- | --- | --- | --- | --- | --- | --- | --- | --- | --- | --- | --- | --- | --- | --- | --- |
| **Patients analyzed, n (%) #** | | 4,805 (90.1) | | 1,781 (89.9) | | 2,036 (93.8) | | 207 (92.8) | | 308 (89.8) | | 124 (70.9) | | 290 (94.2) | | <0.001* | |
| **Outpatient visits, n (%)** | | 4,447 (92.5) | | 1,622 (91.1) | | 1,943 (95.4) | | 152 (73.4) | | 292 (94.8) | | 109 (87.9) | | 271 (93.4) | | <0.001*^&^ | |
| Median (Q1, Q3) | | 3 (2, 5) | | 3 (2, 5) | | 3 (2, 6) | | 2 (1, 7) | | 3 (2, 5) | | 2 (1, 4) | | 3 (2, 6) | |  | |
| Visits/per 100 patients year | | 14.6 | | 11.7 | | 18.3 | | 12.8 | | 13.3 | | 9.2 | | 16.7 | |  | |
| **Emergency room visits, n (%)** | | 1,032 (21.5) | | 474 (26.6) | | 424 (20.8) | | 21 (10.1) | | 41 (13.3) | | 24 (19.4) | | 45 (15.5) | | 0.027*^&^ | |
| Median (Q1, Q3) | | 1 (1, 2) | | 1 (1, 2) | | 1 (1, 2) | | 1 (1, 2) | | 1 (1, 3) | | 1 (1, 2.25) | | 1 (1, 2) | |  | |
| Visits/per 100 patients year | | 1.4 | | 1.4 | | 1.6 | | 0.6 | | 1.1 | | 1.4 | | 1.3 | |  | |
| **Hospitalizations, n (%)** | | 1,947 (40.5) | | 996 (55.9) | | 573 (28.1) | | 161 (77.8) | | 51 (16.6) | | 15 (12.1) | | 148 (51.0) | | <0.001*^&^ | |
| Median (Q1, Q3) | | 1 (1, 1) | | 1 (1, 2) | | 1 (1, 1) | | 1 (1, 1) | | 1 (1, 2) | | 1 (1, 1.5) | | 1 (1, 1) | |  | |
| Visits/per 100 patients year | | 2 | | 2.6 | | 1.6 | | 3.2 | | 0.9 | | 0.6 | | 2.65 | |  | |

The presence of each feature was analyzed until the end of the LPC or LAPC stage. Cryotherapy and HIFU were not included due to the low number of patients (5 and none, respectively). Median (Q1, Q3) is preferred over mean (SD) for interpretation as the feature is non-normal. The Chi-square test was used, except where indicated by (^&^), in which case the Kruskal–Wallis test was applied.* Differences were considered statistically significant when p < 0.05. Adjustment of p-values for multiple comparisons was made using the Benjamini & Hochberg method. PC: prostate cancer; LPC/LAPC: localized PC/locally advanced PC

# Only patients with visits from the beginning to the end of the stage are analyzed.

Cryotherapy and HIFU were not included as specific subgroups due to the low number of patients (5 and none, respectively) and were included in “Other”. Cases included in “Other” category are fully detailed in Table S3.

Supplemental Table 7. Healthcare Resource Utilization Related to Prostate Cancer During Localized Stage.

|  | **Selected LPC/LAPC**  **n=5331** | **Non-Selected LPC/LAPC**  **n=9103** | **p-value** | **Total LPC/LAPC**  **n = 14,434** |
| --- | --- | --- | --- | --- |
| Patients analyzed, n (%) # | 4805 (90.1) | 6349 (69.7) | <0.001* | 11,154 (77.3) |
| Outpatient visits, n (%) | 4447 (92.5) | 5491 (86.5) |  | 9,938 (89.1) |
| Median (Q1, Q3) | 3 (2, 5) | 4 (2, 7) | 0.001*^&^ | 3 (2, 6) |
| Visits/per 100 patients year | 14.6 | 4.2 |  | 5.8 |
| Emergency room visits, n (%) | 1032 (21.5) | 1928 (30.4) |  | 2,960 (26.5) |
| Median (Q1, Q3) | 1 (1, 2) | 1 (1, 2) | <0.001*^&^ | 1 (1, 2) |
| Visits/per 100 patients year | 1.4 | 0.6 |  | 0.7 |
| Hospitalizations, n (%) | 1947 (40.5) | 1737 (27.4) |  | 3,684 (33.0) |
| Median (Q1, Q3) | 1 (1, 1) | 1 (1, 2) | <0.001*^&^ | 1 (1, 2) |
| Visits/per 100 patients year | 2.0 | 0.4 |  | 0.6 |

The presence of each feature was analyzed until the end of the LPC or LAPC stage. PC: prostate cancer; LPC/LAPC: localized PC/locally advanced PC

# Only patients with visits from the beginning to the end of the stage are analyzed.

Fisher's Exact Test for Count Data (two.sided) was used except for ^&^ where Welch Two Sample t-test (two.sided) was used.

Supplemental Table 8. Distribution of follow up time among patients included in the outcome analysis.

|  | **Selected LPC/LAPC**  **N = 5,331** | **2014**  **N= 1226** | **2015**  **N= 1225** | **2016**  **N= 1223** | **2017**  **N= 1031** | **2018**  **N=626** |
| --- | --- | --- | --- | --- | --- | --- |
| Median follow-up (Q1-Q3), years | 2.3 (1.0-3.3) | 4.0 (3.2-4.4) | 3.0 (2.4-3.4) | 2.1 (1.6-2.5) | 1.1 (0.7-1.5) | 0.4 (0.2-0.6) |
| Patients with follow up > median follow up, n (%) | 2586 (48.5) | 1075 (87.7) | 973 (79.4) | 538 (44.0) | 0 (0.0) | 0 (0.0) |
| Patients with follow up > 1 year, n (%) | 3982 (74.7) | 1154 (94.1) | 1123 (91.7) | 1122 (91.7) | 583 (56.5) | 626 (11.7) |
| Patients with follow up > 2 years, n (%) | 2856 (53.6) | 1093 (89.2) | 1046 (85.4) | 717 (58.6) | 0 (0.0) | 0 (0.0) |
| Patients with follow up > 3 years, n (%) | 1584 (29.7) | 992 (80.9) | 592 (48.3) | 0 (0.0) | 0 (0.0) | 0 (0.0) |
| Patients with follow up > 4 years, n (%) | 612 (11.5) | 612 (49.9) | 0 (0.0) | 0 (0.0) | 0 (0.0) | 0 (0.0) |

PC: prostate cancer; LPC/LAPC: localized PC/locally advanced PC

Supplemental Figure 1. UpSet plot of applied filters and resulting patient subsets


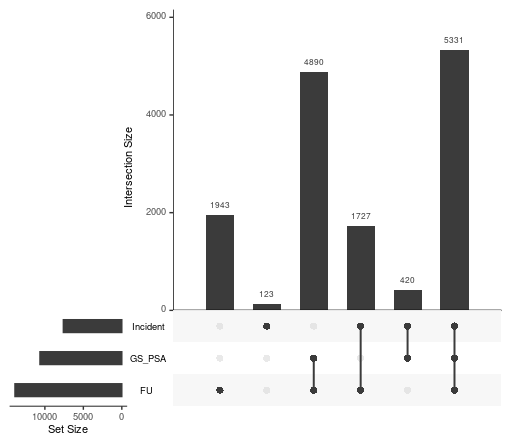


**Figure S1. UpSet plot of applied filters and resulting patient subsets.** This plot illustrates the number of patients meeting different combinations of three key inclusion criteria used for outcome analyses: Incident (incident diagnosis of LPC/LAPC within the study period), GS_PSA (availability of both Gleason score and PSA at index), and FU (availability of follow-up data after diagnosis). The vertical bars show the size of each intersection, while the matrix below indicates the specific combination of filters applied. The largest intersection (far right, 5,331 patients) corresponds to patients meeting all three criteria and represents the final cohort used for outcome analysis. The horizontal bars represent the total number of patients meeting each individual criterion.

Note: As patients could meet one or more criteria simultaneously, intersections are not mutually exclusive.

LPC/LAPC: localized prostate cancer/locally advanced prostate cancer; GS: Gleason; PSA: prostate specific antigen; FU: follow up

Supplemental Figure 2. Event free survival using Kaplan-Meier method for patients with prostate cancer


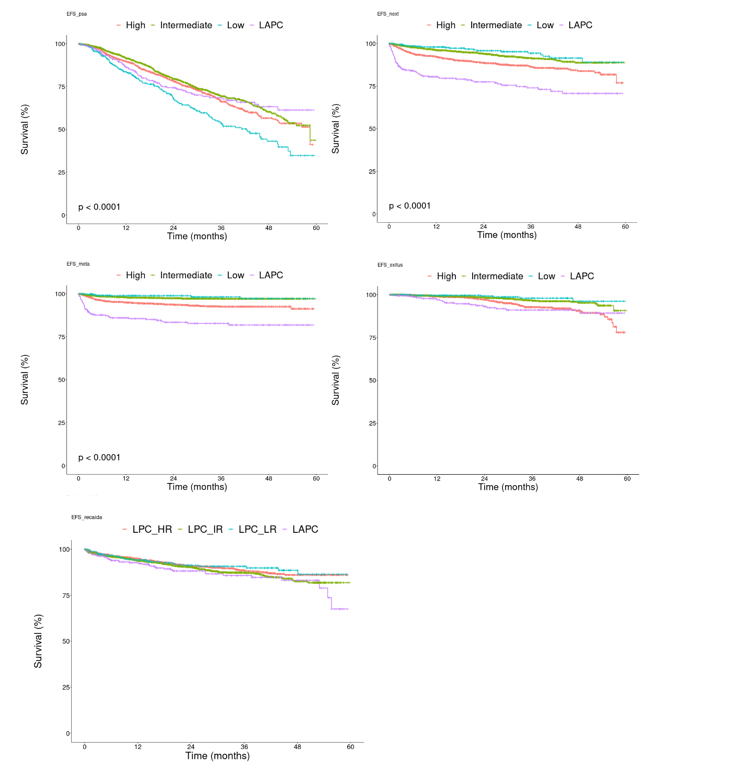


**A**

**B**

**C**

**D**

**E**

**Figure S2.** Event free survival using Kaplan-Meier method for patients with prostate cancer stratified by risk and independently evaluating PSA failure (A), transition to another stage (B), metastatic progression (C), death (D), or recurrence (E) considered for the definition of event.

PC: prostate cancer; LPC/LAPC: localized PC/locally advanced PC; LR: low risk; IR: intermediate risk; HR: high risk.

# References

1. A.V. D'Amico. Risk-based management of prostate cancer, N Engl J Med. 2011;365(2):169-71. doi: 10.1056/NEJMe1103829.

2. L. Canales, S. Menke, S. Marchesseau, A. D'Agostino, C. Del Rio-Bermudez, M. Taberna, J. Tello. Assessing the performance of clinical natural language processing systems: Development of an evaluation methodology, JMIR Med Inform. 2021;9(7):e20492. doi: 10.2196/20492.
